# Facilitating Veterans Health Administration Primary Care for Transitioning Servicemembers: a Novel Virtual Care Clinic

**DOI:** 10.1007/s11606-023-08192-6

**Published:** 2023-06-20

**Authors:** Sumit R. Kumar, Matthew R. Augustine, Rachel L. Sherman, Julie A. Thysen, Meenakshi Zaidi, Daniel T. Gorman, Joseph C. Geraci

**Affiliations:** 1 James J. Peters VA Medical Center, Bronx, NY USA; 2 Present Address: Columbia University Vagelos College of Physicians and Surgeons, New York, NY USA; 3 Present Address: Icahn School of Medicine at Mount Sinai, New York, NY USA; 4 Teachers College, Columbia University, New York, NY USA

## BACKGROUND/OBJECTIVE

Of the 200,000 servicemembers transitioning out of military service annually, many experience psychosocial stressors and high rates of suicide in the first year of transition yet face gaps in accessing care at the Veterans Health Administration (VHA).^1–5^ VHA care can have a protective effect against suicide.^1,2,6^ Our objective was to pilot a novel clinic to rapidly integrate transitioning servicemembers and new veterans (TSMVs) into VHA through virtual primary care.

## METHODS

Our TSMV Virtual Care Clinic was located at the James J Peters VA Medical Center in the Bronx, New York (Bronx VA). The Virtual Care Clinic integrated VHA telehealth, primary care, and mental health. The pilot phase ran from February to June 2022.

Recruitment occurred through the Veterans Sponsorship Initiative (VSI), a program with the Department of Defense (DoD), VHA, and nonprofits that reintegrates TSMVs into civilian life with an emphasis on suicide prevention and enrollment into VHA care. VSI enrolled 269 patients while in active duty from April 2020 to December 2021 and scheduled to exit service prior to May 2022. Of the 269 patients, 171 opted for VHA primary care. Ten patients were enrolled in the Virtual Care Clinic if they (1) had no VHA primary care appointment scheduled at a local VHA facility by February 2022 or later and (2) opted for a virtual video visit.

**Figure 1:**
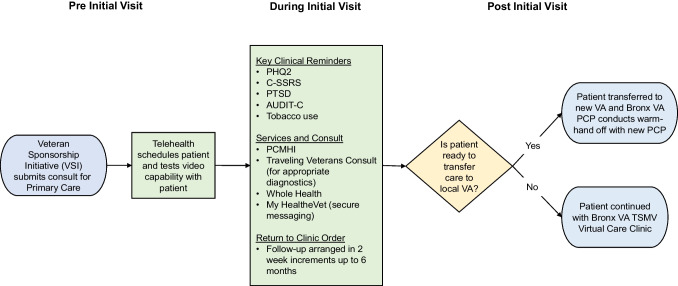
Workflow framework for Bronx VA TSMV Virtual Care Clinic. Shown are the key workflow tasks during the pre, during, and post initial visit phases of care.

Visits were conducted virtually by a primary care physician (PCP) one half day per week with 1-h appointments for new visits. In-person diagnostic testing and specialty consultation were arranged at local VHA facilities through the traveling veteran coordinated care consult. Prescriptions were mailed by the Bronx VA pharmacy. Patients were offered enrollment in a secure messaging application and a consult with VA Whole Health, an integrative health program. Follow-up was maintained until the patient geographically stabilized and care could be transitioned to a local VHA clinic (Fig. 1).

We performed a retrospective chart review to describe the patient characteristics, diagnoses, and services used. Program implementation and analysis received approval by the Bronx VA quality improvement director.

## KEY RESULTS

A total of 10 transitioning servicemembers received primary care in the TSMV Virtual Care Clinic. Two of these patients had received prior VHA care but were lost to follow-up and unable to re-establish VHA care at their local facility. The mean age was 37 years (SD 10.1) and 30% were female. Race/ethnicity was reported as 40% Black/African American and 60% White. All patients were Army veterans who had enlisted, and 80% had exposure to airborne hazards and open burn pits. Patients had a mean service connection disability rating of 70% (SD 40). Patients were located across seven states. Ninety percent of patients had a prior mental health diagnosis. At initial appointment, 70% screened positive for post-traumatic stress disorder (Primary Care PTSD Screen), 50% for depression (Patient Health Questionnaire-2), and no patients screened positive for suicide (Columbia Suicide Severity Rating Scale), alcohol use (Alcohol Use Disorders Identification Test – Concise), or tobacco use. One patient screened positive for military sexual trauma. Of patients with established mental health diagnoses, 60% required refills of psychotropic medications. No patients required urgent or emergent mental health care. Other established diagnoses prior to the first visit are listed in Table 1. After the initial visit, patients used the following VHA services: secure messaging application (90%), pharmacy prescription mailing (60%), mental health (70%), VA Whole Health (70%), and travelling veterans coordinated care (80%).

**Table 1:** Medical History of the Patients Enrolled in the Bronx VA Transitioning Servicemembers and New Veterans Virtual Care Clinic

Medical history — no. (%)^†^	Patients (*n* = 10)
Mental health diagnosis	9 (90)
Elevated blood pressure	5 (50)
Diabetes	1 (10)
Dyslipidemia	4 (40)
Obesity	5 (50)
Musculoskeletal diagnosis	10 (100)
Obstructive sleep apnea	3 (30)
Migraines	6 (60)

^†^Mental health diagnosis: includes any mental health diagnoses (PTSD, adjustment disorder, anxiety, depression, substance use disorders) made prior to initial primary care appointment. Elevated blood pressure: no veteran had a diagnosis of hypertension on initial appointment, elevated blood pressure without diagnosis of hypertension is defined by at least one reading in the past 2 years that was > 130/80 mmHg but not documented as hypertension. Diabetes: defined as hemoglobin A1c ≥ 6.5%. Dyslipidemia: two patients had low-density lipoprotein cholesterol (LDL-C) ≥ 190 mg per deciliter (mg/dL) and were not on statin therapy, two other patients had either LDL-C ≥ 130 mg/dL, high-density lipoprotein cholesterol < 40 mg/dL, or triglycerides ≥ 200 mg/dL. Obesity: defined as any body mass index 30 and over. Musculoskeletal diagnosis: includes any diagnosis involving the shoulder, elbow, wrist, hand, back, hip, knee, ankle, or foot. Obstructive sleep apnea: defined as sleep study with apnea-hypopnea index ≥ 5. Migraines: included patients with documented migraines with treatment during time in service

At the end of the pilot phase, 50% of patients were transitioned to their local VHA. A warm handoff was completed with new PCP via email and *Microsoft Teams* messaging. The remainder continued care through the Virtual Care Clinic.

## DISCUSSION/CONCLUSIONS

The Bronx VA TSMV Virtual Care Clinic delivered accessible and comprehensive primary care nationwide to a diverse population of patients with a significant burden of mental illness. Policies should streamline enrollment and eligibility processes into virtual care clinics to increase access to VHA care by new veterans. Existing services should continue to be leveraged to overcome inter-VHA silos, including pharmacy mailing services and traveling veterans coordinated care to facilitate local diagnostic studies and specialty care. This model demonstrates a scalable opportunity to bridge the care gap between the DoD and VHA for this high-risk population.

## Data Availability

The data used for this concise research report are not publicly available.
